# MIRO: guidelines for minimum information for the reporting of an ontology

**DOI:** 10.1186/s13326-017-0172-7

**Published:** 2018-01-18

**Authors:** Nicolas Matentzoglu, James Malone, Chris Mungall, Robert Stevens

**Affiliations:** 1 School of Computer Science, University of Manchester, Oxford Road, Manchester, UK; 2 FactBio, Innovation Centre, Cambridge Science Park, Cambridge, CB4 0EY UK; 3 Lawrence Berkeley National Laboratory, Berkeley, USA

**Keywords:** Ontologies, Reporting guidelines, Minimum information, Ontology reporting

## Abstract

**Background:**

Creation and use of ontologies has become a mainstream activity in many disciplines, in particular, the biomedical domain. Ontology developers often disseminate information about these ontologies in peer-reviewed ontology description reports. There appears to be, however, a high degree of variability in the content of these reports. Often, important details are omitted such that it is difficult to gain a sufficient understanding of the ontology, its content and method of creation.

**Results:**

We propose the *Minimum Information for Reporting an Ontology* (MIRO) guidelines as a means to facilitate a higher degree of completeness and consistency between ontology documentation, including published papers, and ultimately a higher standard of report quality. A draft of the MIRO guidelines was circulated for public comment in the form of a questionnaire, and we subsequently collected 110 responses from ontology authors, developers, users and reviewers. We report on the feedback of this consultation, including comments on each guideline, and present our analysis on the relative importance of each MIRO information item. These results were used to update the MIRO guidelines, mainly by providing more detailed operational definitions of the individual items and assigning degrees of importance. Based on our revised version of MIRO, we conducted a review of 15 recently published ontology description reports from three important journals in the Semantic Web and Biomedical domain and analysed them for compliance with the MIRO guidelines. We found that only 41.38% of the information items were covered by the majority of the papers (and deemed important by the survey respondents) and a large number of important items are not covered at all, like those related to testing and versioning policies.

**Conclusions:**

We believe that the community-reviewed MIRO guidelines can contribute to improving significantly the quality of ontology description reports and other documentation, in particular by increasing consistent reporting of important ontology features that are otherwise often neglected.

## Background

The need for a common understanding of the entities in a field of interest has led to the widespread adoption of ontologies as a means of representing knowledge [1]. This is particularly true in biology, medicine and healthcare [1, 2]. We also see the use of semantic technologies, including ontologies, increasing outside research in areas such as business and commerce; see, for example, the list of PoolParty customers [3]. Ontologies attempt to represent our knowledge such that inclusion of an entity in a category can be recognised by both humans and computers, for example by using automated reasoners. The definitions and descriptions of every entity in a category may be done in the form of natural language or logical axioms that describe the relationship of one category of objects to objects in another category [4]. Groups of data annotators use ontologies to describe entities; committing to use that ontology seeks to facilitate a common understanding of entities across data sources [1].

Several journals regularly publish ontology description reports (ODR), for example, the Semantic Web Journal (SWJ), the Journal of Web Semantics (JWS) and the Journal of Biomedical Semantics (JBMS). An ODR, in the sense of the current focus of our work, is a published, peer-reviewed report regarding the development of a single ontology represented in a formal language such as the Web Ontology Language (OWL) [5]. Descriptions of an ontology need not, however, be restricted to traditional papers. The original motivation for developing the MIRO guidelines comes from the perceived inadequacy of ODR in the form of published papers, but the use of the MIRO guidelines need not be restricted to traditional ODR. The traditional paper is currently the main route for reporting about an ontology. This should not be the case and, just as research publishing is diversifying in its form, such as through the growth of preprint archives and moving beyond facsimiles of printed paper publishing [6], so should the documentation of an ontology. An ontology itself can and should be the vehicle that disseminates information about its development and status. Indeed, it is plausible that at least some of the reporting could be automated and incorporated in the form of annotations of the ontology. An ontology could, for instance, carry its own descriptions as part of its annotations, and other types of documentation should also carry descriptions of the ontology. This is already the case for datasets that choose to use the W3C’s Vocabulary of Interlinked Datasets (VoID)) [7] which describes metadata about RDF datasets and can be published alongside those datasets to act as a bridge between the publishers and users of data.

While ODRs certainly vary in scope, there is also a high degree of commonality with respect to the process of ontology engineering. Some commonly recurring aspects of the engineering process are, for example, the necessity for some form of knowledge elicitation, formalisation and evaluation. Moreover, there are some commonly shared attributes of the ontology itself: Every ontology has a size, a degree of coverage and a name. As the goal of ontology authors is usually to publish the ontology for community use, another important branch of information items relates to the publishing process, such as licenses, details about the versioning and location on the web.

Unfortunately, and perhaps surprisingly, given that ontologies are a shared conceptualisation about a domain, there is no standard or common understanding about what should be reported in a good, meaningful ODR or other documentation. As a consequence, the contents of published articles appear highly inconsistent. This can be a problem for a variety of reasons. Reviewers for journals and conferences faced with large numbers of ODRs have no reliable guidelines to help them assess the quality of the reports making consistent reviewing between reviewers harder.

There are a large number of (domain-specific) agreements for example when it comes to novel algorithmic contributions (the necessity of reporting a performance benchmark, etc.) but it is still largely unclear how to distinguish reliably between “Here is my ontology” ODRs and substantial contributions that constitute an advancement, such as in modelling, usage or scope, to their respective community. Another problem concerns the ability of a potential consumer in understanding what an ontology is intended to capture. Since traditional ODRs often serve as the main documentation of an ontology, they are often important in building trust among potential users and outlining the intended applicability of an ontology, as merely looking at the ontology may be misleading for a variety of reasons (e.g. intended use, degree of completeness, etc.).

In ontology building, reproducibility is probably a somewhat unrealistic goal; given the same motivation and community, it is unlikely the exact same ontology be produced as a result. Knowing the motivation, the community of interest, the requirements gathered for the ontology, whence the knowledge came to put in the ontology, the axiom patterns used, testing, evaluation, and so on would, however, appear *a priori* to be reasonable features to know about an ontology’s development, along with aspects such as numbers of classes and so on.

Languages such as OWL have annotation properties that support some aspects of ontology description. Editors such as Protégé [8] enable vocabularies such as Dublin Core [9] to be imported so that the ontology and its entities may have dates, creators, descriptions and so on supplied as part of the ontology. Such metadata are, however, insufficient to report on an ontology. Outside research articles describing ontologies, prior work in describing ontologies has been in the area of ontology libraries (registries, repositories and so on) [10–12] where metadata is primarily used for discovery and associated characterisation. The Ontology Metadata Vocabulary (OMV) [10] and the Metadata for Ontology Description and Publication (MOD) [11] are both ontologies that capture aspects of reporting about an ontology. Both seek to promote ontology discovery and re-use. MOD incorporates many aspects about how an ontology was developed within its metadata, such as method, principal classes, and so on and used an open-ended questionnaire to gather material. MOD is more extensive than the OMV, though its primary purpose is still discovery motivated by promoting re-use.

The Minimum Information for the Reporting of an Ontology (MIRO) guidelines aim to guide that which is reported in narratives reporting an ontology such as ODR, as well as other documentation. As such, MIRO is likely to be more extensive than these vocabularies for ontology metadata, despite covering some of the same topics.

We take a broader perspective than discovery, looking at what needs to be reported about an ontology such that its development and status can be understood. Just as the MOD provides more than the OMV, we expect that the MIRO extends the content of the MOD. Our objective is not to create a new ontology, but to establish a set of guidelines for that which should be reported about an ontology. These guidelines may be captured in an ontology itself or other documentation, but the main purpose of MIRO is for use to guide authors and reviewers of papers about ontologies.

The main contributions of this paper are as follows:

- The *Minimum Information for the Reporting of an Ontology* (MIRO) guidelines, which have the aim of improving the quality and consistency of the information content of ontology descriptions.
- A survey with more than 100 respondents to evaluate and refine the guidelines. We present the results of this survey and use participant ratings to prioritise information items by importance.
- A systematic review of the compliance of recent, high-quality ODR published as papers with the MIRO guidelines.

Given the prominence of ontologies in the bio-health community, the MIRO guidelines are of particular interest; they are, however, of general applicability to any ontology description.

## Materials and methods

### MIRO guideline development

The first version of the MIRO was written by a team of three ontology experts (authors of this paper, except Matentzoglu). All three experts have extensive knowledge of building ontologies, reviewing and authoring ODRs, managing ontology collections such as the OBO foundry [13] and organising international ontology-related conferences and workshops such as Semantic Web Applications and tools for the Life Sciences, the International Conference on Biomedical Ontology and the Bio-Ontologies SIG at the Intelligent Systems for Molecular Biology conference. One of the motivations for producing the guidelines stemmed from the difficulty of setting reviewing standards when acting as conference chairs.

Apart from extensive expert knowledge in the ontology domain, the original MIRO information items also took input from reviews of existing ontology metadata vocabularies, the OBO principles [14], and fruitful discussions at events, such as the yearly Ontology Summit [15] and the UK Ontology Network [16].

After gathering an informal list of best practices on reporting, the three experts reviewed each of them internally and organised them into cognate sections, which resulted in the first MIRO draft.

### Survey on the importance of ontology reporting information items

The first draft of the MIRO guidelines was offered to a broad community of ontology paper authors, reviewers, developers and users via a typeform survey [17]. Broadly, the survey had seven sections: (1) basic ontology facts such as the name and URL, (2) motivation for why the ontology was being developed, (3) scope, requirement and community for which the ontology was being developed, (4) knowledge elicitation around how the knowledge included was extracted, (5) ontology content describing technical facts of the ontology such as number of classes, properties, (6) managing change on how the ontology is maintained, and, (7) quality assurance around testing and evaluation.

In this survey, we presented (1) the information item, such as “Ontology name” or “Ontology evaluation”, (2) a Likert scale between 1 (unimportant) and 5 (very important) to rate the subjective relative importance of each item to the respondent and (3) a comment field. Importantly, we did not provide a detailed operationalisation of the MIRO items, i.e. details on how we envisioned a particular information item to be realised in a given ODR or other documentation. We did this for two reasons: (1) We wanted to provide an opportunity for the community to present their position on how a particular item should be realised without too much upfront bias; (2) Some of the items, such as “Testing” and “Evaluation” can mean significantly different things across cases. As we wanted to avoid the impression that we only specified certain cases, we did not include descriptions of how a guideline would be operationalised. In addition to comment fields on each item, we asked, for every section of MIRO, which items or aspects of items were of particular importance to the respondent. Our goal was to create a more detailed characterisation of important items and to use this information to further specify the operationalisation of information items in the final MIRO guidelines, in particular, to emphasise important details in the item description. Towards the end of the survey, we asked the respondents for the single most important criterion when deciding on whether to use an ontology.

Participants viewed ontologies and ontology papers from a variety of perspectives for which different information items may be important to different degrees. To account for these differences in our analysis, we asked participants to indicate their main roles (multiple roles were permitted), i.e. ontology developer, ontology user, reader of papers on ontologies and reviewer of papers on ontologies. We furthermore collected information on the respondents’ professional background, i.e. whether they were student, academic employee, public sector/not-for-profit employee, private sector employee or “Other”.

The questionnaire was sent to email lists read by a wide variety of actors involved with ontologies that would have an interest in how ontologies are reported. Email lists were not limited to only those used by biological and medical ontology developers and users, but to a range of lists reaching a range of domains and technologies. The lists used were:

- The Protéǵe User email list protege-user@mailman.stanford.edu.
- The Open Biomedical Ontologies Discussion list obo-discuss@lists.sourceforge.net.
- The Health Care and Life Sciences Semantic Web Discussion list public-semweb-lifesci@w3.org.
- The Semantic Web email list semantic-web@w3.org.
- The Web Ontology Language email list public-owled@w3.org.
- The UK Ontology Network email list ontology-uk@googlegroups.com.
- The European Ontology Network email list euon@googlegroups.com.
- The European Bioinformatics Institute ontology mailing list ontology@ebi.ac.uk.

After the survey had closed, we analysed the results in the following way:

- We calculated descriptive statistics for the importance ratings given to each item.
- We coded the comments for each item to determine information items of particular interest to respondents and elicit information items potentially beyond the current coverage of MIRO. The comments were coded in a bottom-up fashion, by first collecting the information items mentioned in each comment in a list, then reconciling potentially redundant terminology and finally grouping the comments into categories.
- We analysed the responses to our question for the single most important criterion in the same way as the comments.

### Systematic review of MIRO compliance

To determine to what extent current high-quality ODRs would have adhered to the MIRO guidelines, we performed a systematic review of MIRO compliance [18]. We selected three important journals that regularly publish high-quality ODR AS : the Semantic Web Journal (SWJ), the Journal of Web Semantics (JWS) and the Journal of Biomedical Semantics (JBMS). We decided to focus on recently published work and restricted our search to papers published between March 2015 and May 2016. This time frame was chosen for convenience, to ensure that our sample contained between 15 and 20 relevant ODRs.

First, we retrieved all research papers (168) published by the three journals within the time frame and had three independent researchers filter out obviously unrelated titles according to the following *inclusion criteria*: (1) ontology description paper and (2) primarily about an ontology and its development (where 1 simply describes an ontology and 2 extends this scope with a description of its development and use) and according to the following *exclusion criteria*: (i) primarily system USING ontology, (ii) review about ontologies, (iii) primarily a use case description (study on how the ontology generated value), (iv) an update or extension of an existing ontology. At this stage, we considered all those papers that were thought potentially relevant by at least one reviewer (36). In the second phase, three independent researchers reviewed the abstracts of the remaining papers, after which 19 papers remained. In the last phase, two independent researchers reviewed the remaining papers in depth, which resulted in the exclusion of another 4 papers. The final set of 15 papers was coded according to the 35 information items of MIRO. All codes except for ontology name and ontology owner, which were coded on a three-point Likert scale (absent, mentioned, explicit)—here, ‘explicit’ means that the description of an information item in the paper was present in the narrative with explicit indicators such as “the motivation for developing this ontology was …”, were coded simply with “absent” and “present”. Note that many information items such as “coverage” or “need” can be addressed in a variety of ways and to varying levels of detail. The goal of this review was not to determine the quality of the papers, which would require a coding granularity covering these aspects, but merely to see whether certain items are covered at all.

## Ontology development reporting guidelines

In the following, we call *MIRO* the document that describes the guidelines, *information item* a particular item in the guidelines such as “Ontology name” or “Ontology coverage” and *section* a block of items that belong to a single cognate category such as “Quality Assurance” or “Motivation”. An information item consists of a (1) label, such as “Ontology name”, (2) a description with a definition and details on the operationalisation, (3) a level of importance using the RFC 2119 keywords often used by the W3C [19] *MUST*, *SHOULD* and *OPTIONAL* and (4) an example or a reference to an example.

The guidelines are divided into reporting areas, each with a list of guidelines. The MIRO guidelines in its current state, 5 March 2017, are presented below. For space reasons, we omit the example text here. It can be found in the official guidelines on GitHub [20]. Information items marked with an asterisk were introduced as a consequence of the survey responses.

### A. The basics

- **A.1 Ontology name** (MUST): The full name of the ontology, including the acronym and the version number referred to in the report.
- **A.2 Ontology owner** (MUST): The names, affiliations (where appropriate) and contact details of the person, people or consortium that manage the development of the ontology.
- **A.3 Ontology license** (MUST): The licence which governs the permissions surrounding the ontology.
- **A.4 Ontology URL** (MUST): The web location where the ontology file is available.
- **A.5 Ontology repository** (MUST): The web location (URL) of the version control system where current and previous versions of the ontology can be found.
- A.6 Methodological framework* (MUST): A name or description of the steps taken to develop the ontology. This should describe the overall organisation of the ontology development process.

### B. Motivation

- **B.1 Need** (MUST): Justification of why the ontology is required.
- **B.2 Competition** (MUST): The names and citations for other ontology or ontologies in the same general area as the one being reported upon, together with a description on why the one being reported is needed instead or in addition to the others.
- **B.3 Target audience** (MUST): The community or organisation performing some task or use for which the ontology was developed.

### C. Scope, requirements, development community (SRD)

- **C.1 Scope and coverage** (MUST): The domain or field of interest for the ontology and the boundaries, granularity of representation and coverage of the ontology. State the requirements of the ontology, such as the competency questions it should satisfy. A visualisation or tabular representation is optional, but often helpful to illustrate the scope.
- **C.2 Development community** (MUST): The person, group of people or organisation that actually creates the content of the ontology. This is distinct from the Ontology Owner (above) that is concerned with the management of the ontology’s development.
- **C.3 Communication** (MUST): Location, usually URL, of the email list and/or the issue tracking systems used for development and managing feature requests for the ontology.

### D. Knowledge acquisition (KA)

- **D.1 Knowledge acquisition method** (MUST): How the knowledge in the ontology was gathered, sorted, verified, etc.
- **D.2 Source knowledge location** (SHOULD); The location of the source whence the knowledge was gathered.
- **D.3 Content selection** (SHOULD): The prioritisation of entities to be represented in the ontology and how that prioritisation was achieved. Some knowledge is more important or of greater priority to be in the ontology to support the requirements of that ontology.

### E. Ontology content

- **E.1 Knowledge Representation language** (MUST): the knowledge representation language used and why it was used. For a language like OWL, indicate the OWL profile and expressivity.
- **E.2 Development environment** (OPTIONAL): The tool(s) used in developing the ontology.
- **E.3 Ontology metrics** (SHOULD): Number of classes, properties, axioms and types of axioms, rules and individuals in the ontology.
- **E.4 Incorporation of other ontologies** (MUST): The names, versions and citations of external ontologies imported into the ontology and where they are placed in the host ontology.
- **E.5 Entity naming convention** (MUST): The naming scheme for the entities in the ontology, capturing orthography, organisation rules, acronyms, and so on.
- **E.6 Identifier generation policy** (MUST): What is the scheme used for creating identifiers for entities in the ontology. State whether identifiers are semantic-free or meaningful.
- **E.7 Entity metadata policy** (MUST): What metadata for each entity is to be present. This could include, but not be limited to: A natural language definition, editor, edit history, examples, entity label and synonyms, etc.
- **E.8 Upper ontology** (MUST): If an upper ontology is used, which one is used and why is it used? If not used, then why not?
- **E.9 Ontology relationships** (MUST): The relationships or properties used in the ontology, which were used and why? Were new relationships required? Why?
- **E.10 Axiom pattern** (MUST): An axiom pattern is a regular design of axioms or a template for axioms used to represent a category of entities or common aspects of a variety of types of entities. An axiom pattern may comprise both asserted and inferred axioms. The aim of a pattern is to achieve a consistent style of representation. An important family of axiom patterns are Ontology Design pattern (ODP) which are commonly used solutions for issues in representation.
- E.11 Dereferencable IRI* (OPTIONAL): State whether or not the IRI used are dereferenceable to a Web resource. Provide any standard prefix (CURIE).

### F. Managing change

- **F.1 Sustainability plan** (MUST): State whether the ontology will be actively maintained and developed. Describe a plan for how the ontology will be kept up to date.
- **F.2 Entity deprecation strategy** (MUST): Describe the procedures for managing entities that become removed, split or redefined.
- **F.3 Versioning policy** (MUST): State or make reference to the policy that governs when new versions of the ontology are created and released.

### G. Quality Assurance (QA)

- **G.1 Testing** (MUST): Description of the procedure used to judge whether the ontology achieves the claims made for the ontology. State, for example, whether the ontology is logically consistent, answers the queries it claims to answer, and whether it can answer them in a time that is reasonable for the projected use case scenario (benchmarking).
- **G.2 Evaluation** (MUST): A determination of whether the ontology is of value and significance. An evaluation should show that the motivation is justified and that the objectives of the ontology’s development are met effectively and satisfactorily. Describe whether or not the ontology meets its stated requirements, competency questions and goals.
- **G.3 Examples of use** (MUST): An illustration of the ontology in use in its an application setting or use case.
- G.4 Institutional endorsement* (OPTIONAL); State whether the ontology is endorsed by the W3C, the OBO foundry or some organisation representing a community.
- G.5 Evidence of use* (MUST): An illustration of active projects and applications that use the ontology.

## Results

We sent our first call for participation in the survey on the MIRO guidelines proposal on 11^th^ April 2016 and closed the survey on 12^th^ May 2016. After two weeks from the announcement, reminder emails were sent out to the selected email lists. There were 110 responses in total to the survey. This large number of responses gives us a good level of confidence of a reasonable representation from the ontology community. The R analysis documentation for the survey data can be found at [21].

### Demographics of responders

**Fig. 1:**
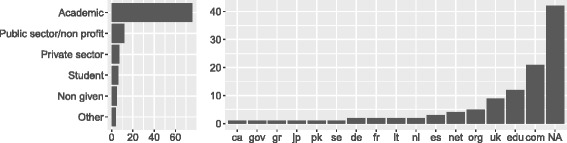
Demographics of respondents. Left: Jobs of respondents, overall counts. One job per respondent. Right: Institutional spread of respondents, overall counts of email top level domain. One email per respondent

**Fig. 2:**
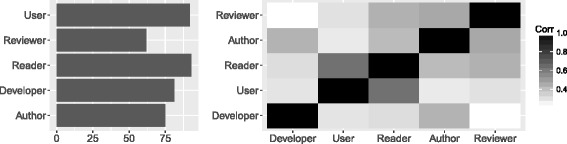
Demographics of respondents. Left: Roles of respondents, overall counts. Multiple roles per respondent. Right: Correlation matrix for roles of users. The darker, the more highly correlated

Figure 1 shows the jobs responders declared. The highest responders were academic employees (76 out of 110) with the second largest group being public sector/not-for-profit employees (12). From the top level domains (TLD) of the email addresses given by the responders, we created a geographical profile (Fig. 1, right). We witnessed a broad spread of TLDs, which indicates that our advertisement strategy made the survey widely visible. Figure 2 shows the roles declared by responders. Almost half of the respondents (44%) reported to act in all 5 roles, with another 12% acting in all roles except *reviewer of ontology papers*. From the correlation matrix on the right, there appear to be roughly three major groups of responders: (1) users and readers, (2) paper authors, reviewers and readers and (3) developers and authors.

### Importance of MIRO information items

**Fig. 3:**
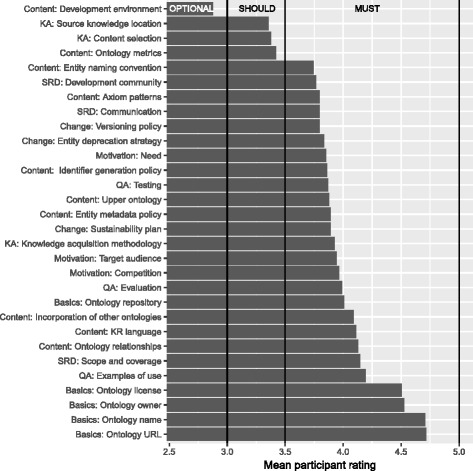
Mean rating for each information item. Vertical lines correspond to importance level (optional, should, must)

Figure 3 shows the mean importance rating given to each MIRO information item. Inspection of the figure shows three major step changes in the importance that we have mapped to categories of importance: features that *must* be given; those that *should* be given; and those that are *optional* as to whether or not they are given. The majority of the MIRO information items are deemed to be mandatory: Only the editor used for creating the ontology (optional), the location of the source knowledge (should), the ontology content selection (should) and the basic ontology metrics (should) were not. That ontology metrics were thought of as having lower importance was surprising to us, as metrics are a relatively simple mechanism for communicating (for example) the scale of the ontology, and complexity of the modelling (for example in the form of a breakdown of axiom type counts, or simple OWL 2 profile memberships) and can usually be automatically computed. Another low priority item was the explanations of how the content was selected, i.e. how the entities and classes were chosen that should be part of the ontology. In practice, they are often implicit in the requirements of the ontology and are perhaps therefore deemed of lower importance.

Table 1 shows the descriptive statistics of all 30 original information items, sorted by standard deviation. The standard deviation can be seen as a measure of disagreement: the lower it is, the more respondents agreed on a rating. It is notable that the standard deviation is strongly negatively correlated (-0.85) with the mean: The higher the average rating, the lower the disagreement. For example, basics such as the URL and the ontology name have very high mean ratings and the lowest standard deviations among all items. For items like ontology metrics, such as class and property counts, and the editing tool with which the ontology was built, receive low mean ratings, and have the highest standard deviations; they are very important to a handful of people but unimportant to others.

**Table 1:** Descriptive statistics of all information items in MIRO (mean, median, standard deviation)

MIRO item	Rank	Mean	Med	SD
Basics: Ontology URL	1	4.72	5	0.68
Basics: Ontology name	2	4.71	5	0.70
Basics: Ontology license	4	4.50	5	0.79
SRD: Scope and coverage	6	4.15	4	0.84
SRD: Development community	25	3.77	4	0.86
Basics: Ontology owner	3	4.53	5	0.87
Content: Ontology relationships	7	4.13	4	0.88
Content: Incorporation of other ontologies	9	4.09	4	0.95
Motivation: Target audience	13	3.94	4	0.96
Content: Axiom patterns	24	3.80	4	0.96
QA: Examples of use	5	4.19	5	0.99
KA: Knowledge acquisition methodology	14	3.93	4	0.99
Content: Entity metadata policy	16	3.89	4	1.02
Content: KR language	8	4.11	4	1.03
Content: Upper ontology	17	3.88	4	1.03
Change: Versioning policy	23	3.80	4	1.03
QA: Testing	18	3.87	4	1.04
KA: Content selection	28	3.38	4	1.04
Content: Entity naming convention	26	3.74	4	1.04
Basics: Ontology repository	10	4.01	4	1.04
Change: Entity deprecation strategy	21	3.83	4	1.07
Motivation: Competition	12	3.96	4	1.07
Motivation: Need	20	3.85	4	1.08
Content: Identifier generation policy	19	3.86	4	1.08
QA: Evaluation	11	3.99	4	1.08
SRD: Communication	22	3.80	4	1.09
Change: Sustainability plan	15	3.89	4	1.09
KA: Source knowledge location	29	3.36	3	1.09
Content: Ontology metrics	27	3.42	3	1.18
Content: Development environment	30	2.88	3	1.30

*Abbreviations*: *SRD* Scope, requirements, development community, *QA* Quality assurance, *KA* Knowledge acquisition, med–Median, *sd* standard deviation

Data is sorted by standard deviation (sd) in order to highlight the items that had the largest disagreement

We have ranked the information items shown in Fig. 3 from 1 to 30, with 1 being the most important feature (i.e. the one that received the highest mean rating) and 30 being the least important. Apart from the overall ranking (see Table 1), we computed the ranking for each user role separately, to find differences in relative importance. We will report this difference in what follows by the difference in rank compared to the overall rank, mentioning only those items that deviate by at least 4 positions in the ranking. For example, authors of ontology papers are less interested in the knowledge acquisition methodology (-7) than the mean. Indeed, if only the scores of respondents that are ontology authors are considered, the MIRO item “knowledge acquisition methodology” would fall 7 places in rank order (from position 14 to 21).

Depending on the role of the respondents, some ratings differed markedly in their overall rank. Apart from the above-mentioned uninterest in the knowledge acquisition methodology, authors of ontology papers are less interested in the identifier generation policy (-6) and the reference to the repository holding the ontology (-4) compared to the overall ranking. On the other hand, they are more interested in upper ontologies (6) and the community that is being engaged to develop the ontology (6) than all of the other groups.

Developers are less interested in reporting about testing (-6), while they care more about the sustainability plan (5) and an entity deprecation strategy (6) than the mean. That ontology developers rank testing so low (rank 24 of 30 items on the developers ranking) is, at least to us, worrying. Testing should be a critical part of the development lifecycle, and reporting on the results of this testing is crucial for increasing confidence in potential users. Reviewers, like authors, find the knowledge acquisition methodology less important than the mean (-6), while they, perhaps surprisingly given their role, ascribed considerably more importance to information on why the ontology is needed (5). Ontology users care less than the rest about which upper ontologies are used (-4) but rank the entity deprecation strategy (7) higher than the mean. Lastly, readers of ontology papers are less interested to learn about the knowledge acquisition methodology (-6) as well as details on the entity metadata policy (-5) compared to the mean, while caring more than the mean about the sustainability plan (5) and the entity deprecation strategy (5) - the latter two items both associated broadly with considerations for planning and risk.

### Analysis of comments

The MIRO as presented in the survey was restricted to very short descriptions, often only a simple label, and did not provide details on the individual information items. In the draft, the authors had provided some operationalisation for the information items. These were omitted from the survey to encourage as many comments about the items as possible. As a result of this feedback, we added four new information items to the original draft (for details, see “Ontology development reporting guidelines” section):

- Methodological framework
- Dereferenceable IRIs
- Institutional endorsement
- Evidence of use

Furthermore, we used the feedback to improve the operationalisation of the MIRO items, as well as perform some minor changes to the item labels.

In the following, we present our somewhat ancillary analysis of the most frequently discussed topics in the comment fields (see “Materials and methods” for details). Note that these categories do not always correspond to MIRO items. The reason for that is that we did not want to deviate too much from the labelling of the responders. Our goal was to capture areas of personal concern to the responders and establish a secondary metric of importance that is orthogonal to our main metric (the ratings) and provides further insight.

As can be seen in Table 2, the information item thought to be most important was that of coverage and scope. Put crudely, this is ‘what does the ontology claim to cover?’ and ‘in what detail is it covered by the ontology?’. This aspect of ontology reporting directly corresponds to the MIRO item “SRD: Scope and coverage”, which is placed sixth in the main ranking (see Table 1); the highest ranking, just after the basics: ontology URL, name, owner and license and “QA: Example of use”. The latter is also the information item that corresponds most closely to the second most important topic, “Use Case”, with 18 mentions. Responders were very interested for the report to reveal exactly the scenario for which the ontology was designed, to decide whether it is likely to fit their own. This interest was further reflected by the topic “Evidence of use”, which subsequently received its own MIRO item; people seem to just want to be reassured that the ontology was not merely developed for its own sake (or purely academic reasons) before they give it their trust and apply it to their own scenario.

**Table 2:** Analysis of the comments on what is most important

Topic	Count
Scope and coverage	23
Use case	18
Active community	16
Content	11
Publishing and life cycle	10
Interoperability	9
Metadata and documentation	8
Representation	8
Evidence for use	7
Usability	5
Other	4

All comments were coded and grouped into topics. The counts on the right are the total number participants mentioning an item belonging to the group

The “active community” topic (third most frequently mentioned topic) corresponds most closely to the MIRO item “SRD: Development community”, which is ranked 25th according to importance. The discrepancy here can be explained by the sentiment of the comments mentioning the “active community”: the emphasis of the comments was on the community being “active”, rather than a description of the creators of the community. The importance of the community being “active” was perhaps not so much regarding the community’s role as an information item in a report, rather than a key selection criterion for whether or not to use the ontology reported upon [22]. We feel that most of this ancillary comment analysis should be viewed in the light of this: often what should be reported and what should *be the case*. These are two very different questions in practice but are perhaps interpreted as the same by our survey responders.

The remaining, slightly less important topics of interest do not exactly correspond to individual MIRO items. “Publishing and life cycle” for example relates to aspects of the MIRO “Managing change” section, as well as other information items such as the URL, the communication infrastructure and the repository. However, they all relate to practical considerations: pieces of information that help users to decide whether or not to employ the ontology. Is it represented in a format I can use (representation)? How easy is it to use in my scenario (Usability)? How good is the ontology document (metadata and documentation)? The topic of “content”, the fourth most frequently mentioned after “active community” is very vague, but most probably expresses the sentiment of the responders that it is important to them what is actually in the ontology; in terms of our issue of what is important to report on, this means that good descriptions of what is represented in the ontology are critical, and–perhaps–often neglected.

### Compliance of existing papers with MIRO guidelines

Table 3 shows the 15 papers that were selected for inclusion into the review process (for methodological details, see “Materials and methods”).

**Table 3:** Reviewed papers

Title	Journal	Year
LOTED2: An Ontology of European Public Procurement Notices [27]	SWJ	2016
PPROC, an Ontology for Transparency in Public Procurement [28]	SWJ	2016
Overview of the MPEG-21 Media Contract Ontology [29]	SWJ	2016
The Document Components Ontology (DoCO) [30]	SWJ	2016
The Data Mining OPtimization Ontology [31]	JWS	2015
My Corporis Fabrica Embryo: An ontology-based 3D spatio-temporal modeling of human embryo development [32]	JBMS	2015
Development of an Ontology for Periodontitis [33]	JBMS	2015
Developing VISO: Vaccine Information Statement Ontology for patient education [34]	JBMS	2015
Development and application of an interaction network ontology for literature mining of vaccine-associated gene-gene interactions [35]	JBMS	2015
The cellular microscopy phenotype ontology [36]	JBMS	2016
The Non-Coding RNA Ontology (NCRO): a comprehensive resource for the unification of non-coding RNA biology [37]	JBMS	2016
OBIB-a novel ontology for biobanking	JBMS	2016
VICO: Ontology-based representation and integrative analysis of Vaccination Informed Consent forms [38]	JBMS	2016
MicrO: an ontology of phenotypic and metabolic characters, assays, and culture media found in prokaryotic taxonomic descriptions [39]	JBMS	2016
Representing vision and blindness	JBMS	2016
Towards exergaming commons: composing the exergame ontology for publishing open game data [40]	JBMS	2016
An ontology for major histocompatibility restriction [41]	JBMS	2016

Journals are Semantic Web Journal (SWJ), Journal of Biomedical Semantics (JBMS) and Journal of Web Semantics (JWS)

*Compliance* is defined as *the number of papers that mention a MIRO item* divided by the *overall number of papers*. We define the following *compliance level* categories: If the compliance is

- <20%, we consider it “very low” (V),
- =20% and <50%, we consider it “low” (L),
- =50% and <80%, we consider it “medium” (M),
- >=80% we consider it “high” (H).

The *rating levels* “optional” (O), “should” (S) and “must” (M) are defined in “Importance of MIRO information items” section. Table 4 shows, for each of the MIRO information items the compliance contrasted with the ratings from the ontology survey and the compliance-ratings factor (CRF). The compliance-rating factor comprises two letters, the first of which corresponds to the rating level and the second to the compliance level. For example, MH stands for a “must” (M) rating with “high” (H) compliance. The first observation to be made is that a large proportion of MIRO items fall under the MH category (13 out of 30, 43.33%). We need to remember, however, that the survey only assessed whether an item was covered at all, so no conclusions can be derived on how well these items were covered by the original papers. The second most important category is ML (“must” rating, “low” coverage) with 5 out of 30 items (16.67%), followed by MM (“medium” coverage) and MV (“very low” coverage) with 4 items (13.33%).

**Table 4:** MIRO items ordered by compliance (COM), including the rating (RAT) from the ontology survey”

MIRO item	RAT	COM	CRF
SRD: Scope and coverage	4.15	100.00	MH
Content: KR language	4.11	100.00	MH
Motivation: Target audience	3.94	100.00	MH
Motivation: Need	3.85	100.00	MH
Content: Axiom patterns	3.80	100.00	MH
Basics: Ontology URL	4.72	93.33	MH
Content: Ontology relationships	4.13	93.33	MH
SRD: Development community	3.77	93.33	MH
Basics: Ontology name	4.71	90.00	MH
QA: Examples of usage	4.19	86.67	MH
Content: Incorporation of other ontologies	4.09	86.67	MH
Motivation: Competition	3.96	80.00	MH
KA: Knowledge acqu. methodology	3.93	80.00	MH
Content: Ontology metrics	3.42	80.00	SH
Content: Development environment	2.88	73.33	OM
QA: Evaluation	3.99	66.67	MM
Content: Upper ontology	3.88	66.67	MM
KA: Content selection	3.38	66.67	SM
Basics: Ontology owner	4.53	53.33	MM
Basics: Ontology repository	4.01	53.33	MM
SRD: Communication	3.80	40.00	ML
Content: Entity metadata policy	3.89	33.33	ML
Basics: Ontology license	4.50	26.67	ML
QA: Testing	3.87	26.67	ML
Content: Entity naming conventions	3.74	26.67	ML
KA: Source knowledge location	3.36	26.67	SL
Content: Identifier generation policy	3.86	6.67	MV
Change: Versioning policy	3.80	6.67	MV
Change: Sustainability plan	3.89	0.00	MV
Change: Entity deprecation strategy	3.83	0.00	MV

The compliance-rating factor (CRF) is described in “Compliance of existing papers with MIRO guidelines” section

We believe the ML and MV categories to be the most important ones to consider, as they represent the highest discrepancy between what readers wish to see in a paper compared to what they would actually find. The four items in the MV category, the identifier generation policy, the versioning policy, the sustainability plan and the entity deprecation strategy all concern aspects of the ontology lifecycle. It is perhaps less surprising that the identifier generation policy and the deprecation strategy are rarely mentioned at all: they may either be taken for granted (perhaps implicitly by referring to the compliance to OBO principles) or simply not be applicable, for example in cases where the scope of the ontology is small and well-defined, which would render the use of identifiers unnecessary, as it would a bespoke deprecation strategy. The other two items, however, versioning and sustainability plan, are applicable to all ontologies, and neglecting to give the reader a sense of them can easily lead to the impression that the development of the ontology is a one-off, zero maintenance, in some cases even throw-away prototype case study. In our opinion, this is a wide-spread problem even beyond the scope of this review, and finds another confirmation in the fact that just about half of the reviewed papers explicitly referred to a versioned repository such as GitHub (53%) and less than half mention something like issue tracking or email lists (40%).

The items in the ML category are the entity metadata policy (33.33% coverage), an explicit mention of the license under which the ontology may be used (26.67%), means of communication such as email lists and issue tracking (40%), an explicit naming convention for entities (26.67%) and an explicit testing strategy (26.67%). Again, most of these metrics concern the management of the ontology life-cycle.

Noteworthy is the low compliance on the testing item. Testing differs from an evaluation in that it is not concerned with the question of whether the ontology in principle does its job (this would be the evaluation, for example through a use case study), but a systematic attempt to capture non-functional aspects of the ontology, such as performance (for example classification time when reasoning is required) or correctness of the hierarchy after modelling or mapping with other ontologies, etc. At the very least, we feel, it should be stated whether or not the ontology is parseable by the usual tools, like the Jena API [23] or the OWL API [24], Protégé or OBO-Edit.

## Discussion

In this paper, we addressed the problem of what to report upon in ‘ontology description’ reports’ and potentially other documentation, including in the ontology itself. There are several actors in this scenario, for example: (1) paper or documentation authors writing up the report that need guidance on what aspects of the ontology development process to cover, (2) ontology users (which, among our survey respondents, frequently coincide with readers of ontology papers) that need guidance for the ontology selection process and (3) ontology developers that are just about to start development that require a checklist for recording the forthcoming development. Many of the respondents to our survey adopt a broad range of ontology-related roles. How ontologies are reported needs to satisfy actors playing all of these roles.

We were gratified by the large number of responses we received to our survey in a relatively small period of time. Even after the survey was formally closed, we kept receiving responses, which suggests that the issue of what should be reported about an ontology is of significant interest in the community. With the 110 responses used in this study, we think the survey is representative of the community; indeed, the number of responses approximates the number of people attending bio-ontology meetings such as the Bio-Ontologies COSI at the ISMB and the International Conference on Biomedical Ontology.

The vast majority of the MIRO guidelines have the importance designation of *must*. This may appear onerous, but the MIRO guidelines are a ‘minimal’ list of that which should be reported. Being ‘minimal’ indicates that the MIRO information items are intrinsically those that are most important. Thus, a claim of compliance with the MIRO guidelines should mean that the ontology is reported well. Besides, our importance designations are driven by the data supplied in the survey; irrespective of any possible response biases [25] and we have trusted the data.

A methodological problem we faced during the paper coding was to judge whether a code was sufficiently covered when it is only implicitly mentioned. For example, items such as scope and need are very hard to *not* cover at all. That is why the compliance of the MIRO items “SRD: Scope and coverage” and “Motivation: Need” was 100% (remember that this does not mean they were covered *well*, only that they were at least mentioned). Coding such items was, however, sometimes challenging as they were not *explicitly* mentioned, for example by saying “The need of this ontology emerged from…’ or “The ontology covers all categories of…’. As reviewers, we would have liked such explicit statements, and it is likely that readers of ontology papers would also benefit from clarity resulting from stating information items explicitly.

The most important categories were around ontology scope and coverage, and this is perhaps unsurprising. Apart from this, a category of very high concern to the community (as reflected for example by “Analysis of comments” section) was the area of publishing and ontology life-cycle related issues. Such issues touch on some MIRO items such as the sustainability plan, versioning policy and repository location. We found that this area is frequently absent in high-end ODR; the three items with the lowest compliance all fall under this category. We believe that in some cases, this may point to the intention of the ontology developers to produce a one-off product rather than produce a continuously maintained knowledge artefact. Our recommendation from this work is that authors should make these parts explicit in the report. If an ODR suggests that it provides an important service, especially if positioning as a reference to be widely adopted by a community, a description of the sustainability plan must be included. However, it is also possible that ontology developers are simply not entirely conscious about how important such aspects are for deciding whether or not to employ an ontology. Most ODR focus on ontology content and knowledge acquisition rather than aspects of the development life-cycle that become relevant only after the first draft of the ontology is published. The MIRO guidelines and the analysis we presented should help to improve awareness regarding such information items and their importance to users.

Community feedback was an integral part of the development process of the MIRO guidelines. Not only were we able to derive categories of importance from the ratings; we were also able to identify four new categories that were not covered by the first MIRO draft. We further used community feedback to improve the definitions and labels of the information items. For the MIRO guidelines to have an impact on the quality of ontology papers, we believe that MIRO and projects similar to it should be community-driven to reach the highest degree of consensus possible, in much the same way as the ontology community has developed some of the most popular ontologies.

In the future, it should also be possible to extend MIRO beyond guidelines for reporting in text to a more structured form. Such a form would enable the metadata reporting MIRO to be accessed programmatically in much the same way as approaches such as VoID [7] have successfully taken. Besides, a W3C working group, similar to that pursued by the VoID authors, to further the establishment of a structured form of MIRO could help MIRO become a more widely adopted method used for publishing ontologies in literature and on the web. Those involved in developing and using ontologies should be involved in such an effort, but an important additional participant would be the maintainers of ontology libraries and repositories such as the OBO Library, BioPortal and the Ontology Lookup Service (OLS). Adoption and publishing of MIRO alongside ontologies in these repositories would be a valuable asset when considering the suitability of an ontology for use.

As well as structured, computationally amenable reporting, it should also be possible to derive some aspects of the MIRO guidelines programmatically. Obvious cases include numbers of entities in an ontology, relationships used, location, licence and location etc. Programmatically extracting axiom patterns is more difficult, but attempts have been made such as extracting syntactic regularities from ontologies as proxies for axiom patterns [26], which finds syntactic regularities in ontologies. With such computational support, creating sound, up-to-date descriptions of an ontology in accordance with the MIRO guidelines becomes easier.

## Conclusions

Appropriate reporting of ontologies and ontology development processes is important for the understanding of those ontologies. To this end, we have created a set of minimum information guidelines for ontology reports upon which we have gathered input from the ontology community. The method we have used to develop the MIRO guidelines give confidence that they are well supported. We learned which information items are of particular importance to the community, and we learned where the current reporting is lacking. The MIRO guidelines need to be an evolving reporting standard, especially with respect to how each of the reporting items is operationalised; we welcome continuous input on the MIRO guidelines [20].

We recommend the MIRO guidelines to both ontology users, authors and reviewers in the ontology community to improve the presentation of their work.

## Abbreviations

CRF: Compliance-ratings factor
IRI: International resource identifier
JBMS: Journal of biomedical semantics
KA: Knowledge acquisition
MIRO: Minimum information for reporting an ontology
MOD: Metadata for ontology description and publication
OWL: Web ontology language
ODP: Ontology description paper
OMV: Ontology metadata vocabulary
OBO: Open biomedical ontologies
QA: Quality assurance
SWJ: Semantic web Journal
SRD: Scope, requirements, development community
TLD: Top level domain
VOID: Vocabulary of interlinked datasets
WSJ: Journal of web semantics
W3C: World Wide web consortium
